# MiR-455-5p Suppresses the Progression of Prostate Cancer by Targeting CCR5

**DOI:** 10.1155/2019/6394784

**Published:** 2019-04-11

**Authors:** Qianwei Xing, Huyang Xie, Bingye Zhu, Zhiwei Sun, Yeqing Huang

**Affiliations:** Department of Urology, Affiliated Hospital of Nantong University, Nantong, Jiangsu Province, China

## Abstract

Accumulated evidence indicates that miR-455-5p functions as tumor suppressor in the progression of various cancers. However, the mechanism through which miR-455-5p influences the tumorigenesis of human prostate cancer (PCa) remains undetermined. In this study, reanalysis of data obtained from the Memorial Sloan Kettering Cancer Center showed that miR-455-5p can be used as biomarker for PCa diagnosis and predictor of poor prognosis. Functional assays indicated that miR-455-5p overexpression could suppress cellular proliferation, inhibit tumor growth, and trigger apoptosis by activating and cleaving caspase 3. We experimentally verified that miR-455-5p negatively regulated the C–C motif chemokine receptor 5 (CCR5). Overall, our data demonstrate that miR-455-5p suppressed PCa cellular proliferation and induced cell apoptosis by downregulating CCR5. Thus, miR-455-5p may be considered a new therapeutic strategy for PCa.

## 1. Introduction

Prostate cancer (PCa) is the most commonly diagnosed cancer among men according to the statistics of the American Cancer Society. Approximately 29,430 deaths caused by PCa were estimated in America in 2018 [1]. PCa deaths are typically caused by metastatic castration-resistant PCa (mCRPC), which develops from androgen-dependent PCa (ADPC) [2]. Statistically, the median survival of men with mCRPC is less than 3 years. Similar to other human cancers, PCa is associated with a wide range of genetic aberrations and abnormal expression. However, the detailed mechanisms underlying the initiation and progression of PCa carcinogenesis remain unclear [2].

Mature microRNA (miRNA) is a kind of small (approximately 22 nucleotides in length), noncoding RNA that negatively regulates over 1/3 of coding genes by binding to the 3′-untranslated regions (3′-UTR) of the genes [3]. To date, numerous miRNAs have been discovered and verified to serve as tumor suppressors or oncogenes in PCa progression. For instance, miR-146a suppresses cell viability and induces cell apoptosis by targeting EGFR, ROCK1, and Rac1 [4–6]. By contrast, low expression of miR-146a in CRPC was believed to cause the malignant transition of PCa from ADPC to CRPC [7].

Published data indicate that miR-455-5p is downregulated in several kinds of cancers and serves as a tumor suppressor in colorectal cancer [8], non-small-cell lung cancer [9], hepatic cancer [10], gastric cancer [11], and oral squamous cell carcinoma [12]. To date, some published articles have primarily explored the expression and precise role of miR-445-5p in PCa [13].

In this study, data focused on miR-455-5p from the PCa database of Memorial Sloan Kettering Cancer Center (MSKCC) were reanalyzed. Results revealed that miR-455-5p expression evidently decreased in PCa compared with that in normal prostate tissues. Patients with high Gleason scores exhibited low mi-455-5p expression, and those with lower miR-455-5p expression exhibited shorter relapse-free survival (RFS) times. The inhibition of miR-455-5p in LNCAP promoted cell viability and colony-formation ability and inhibited apoptosis in PCa. By contrast, overexpression of miR-455-5p in DU145 inhibited cell viability and colony-formation ability and promoted apoptosis in PCa. The stably overexpressed miR-455-5p suppressed tumor growth in vivo. We then verified that miR-455-5p could suppress the PCa cell progression by directly targeting CCR5. Data from PCa database suggested an inverse correlation of expression between miR-455-5p and CCR5. And in contrast to miR-455-5p, PCa patients with high CCR5 expression showed shorter RFS times. These findings imply that miR-455-5p serves as a tumor suppressor by targeting CCR5 in PCa and could be exploited as a new therapeutic target.

## 2. Materials and Methods

### 2.1. Data Mining and Bioinformatics Analysis

The miR-455-5p expression levels in the PCa tissues were retrieved from the MSKCC PCa database (GSE21032, http://www.ncbi.nlm.nih.gov/geo/query/acc.cgi?acc=GSE21032) [14]. Clinical patient data (miR-455-5p and CCR5) were downloaded from the MSKCC database (http://www.mskcc.org). Data on the miR-455-5p and CCR5 expression among normal prostate tissues and PCa tissues, as well as the corresponding biochemical relapse-free time after radical prostatectomy, were reanalyzed from the database. Receiver-operating characteristic (ROC) curves were generated, and the area under the curve (AUC) was considered to evaluate the sensitivity and specificity of the use of miR-455-5p expression to diagnose PCa.

### 2.2. Cell Culture and Transfection

One normal prostate cell line RWPE1 and three human PCa cell lines (LNCAP, DU145, and PC3) were obtained from Shanghai Cell Bank, Chinese Academy of Sciences. These cells were cultured in RPMI 1640 (Hyclone, Beijing, China) supplemented with 50 U/ml penicillin, 50 mg/ml streptomycin (Hyclone, Beijing, China), and 10% fetal bovine serum (Gibco, Gaithersburg, Maryland, USA) at 37°C in an atmosphere of 5% CO_2_. MiR-455-5p/negative-control mimics (miR-NC) and miR-455-5p inhibitor/inhibitor-NC were purchased from GenePharma (Shanghai, China), and si-CCR5/si-NC were purchased from Santa Cruz Biotechnology (California, USA). The overexpression vector for human CCR5 (pcDNA3.1-CCR5) was purchased from YouBio Technology (Nanjing, China). An empty vector was used as negative control. Transfections with oligonucleotides or plasmid vectors were performed with Lipofec-tamine-2000 (Invitrogen, Carlsbad, New Mexico, USA). The sequences of miR-455-5p and inhibitor oligonucleotides were as follows:

  miR-455-5p mimics: 5′–GCAGUCCAUGGGCAUAUACAC–3′,
  miR-NC: 5′–UUCUCCGAACGUGUCACGUTT–3′,
  miR-455-5p inhibitor: 5′–GUGUAUAUGCCCAUGGACUGC–3′ and
  inhibitor-NC: ACGUGACACGUUCGGAGAATT.

### 2.3. RNA Isolation and Quantification by Real-Time PCR

Total RNA was extracted from tissues or cells using TRIzol (Invitrogen, Carlsbad, New Mexico, USA) in accordance with the manufacturer's instructions. RNA concentration was measured using NanoDrop2000c (Thermo Scientific, Wilmington, Delaware, USA). Quantitative reverse-transcription PCR (qRT-PCR) reactions for miR-455-5p were performed using the SYBR Green PCR Master Mix of Hairpin-miRNA RT-PCR Quantitation Kit (GenePharma, Shanghai, China) in accordance with the manufacturer's protocols as previously described [15].

### 2.4. CCK-8 Assay

Cells were transfected with oligonucleotides and cultured overnight. Subsequently, the cells were trypsinized and seeded at 2000 cells/well in a 96-well plate. Cell viability was measured using the CCK-8 assay kit (Beyotime, Shanghai, China) in accordance with the manufacturer's instructions as previously described [15].

### 2.5. Colony-Formation Assay

A total of 500 cells were seeded after transfection into six-well plates and cultured for 9 days. The colonies were fixed with formalin (10%) and stained with crystal violet on the 10th day. The colonies that exceeded 50 cells per colony were counted.

### 2.6. Evaluation of Apoptosis by Flow Cytometry

Apoptosis was analyzed by the annexin V-FITC and propidium iodide (PI) staining method as previously described [15]. The treated cells were analyzed by flow cytometry (Becton Dickinson, Franklin Lakes, New Jersey, USA). The experiments were performed in triplicate.

### 2.7. Western Blot Analysis

Cells were lysed by RIPA buffer (Beyotime, Shanghai, China) supplemented with protease inhibitors. Protein concentration was measured using the BCA assay (Beyotime) in accordance with the manufacturer's instructions. Total protein was electrophoresed by SDS-PAGE. The proteins were then transferred to a polyvinylidene fluoride membrane (Millipore, Billerica, MA, USA) and blocked for 1 h with 5% skim milk at room temperature. Incubation with primary antibodies was performed overnight at 4°C. The blots were incubated with horseradish peroxidase (HRP)-labeled secondary antibodies, and the signal was detected using ECL (Beyotime). The following antibodies were used for Western blot: rabbit anti-GAPDH (1:500; Beyotime, Shanghai, China), rabbit anti-CCR5 (1:1000; Protein Tech, Wuhan, China), rabbit anti-caspase 3 (1:1000; Protein Tech, Wuhan, China), rabbit anti-cleaved caspase 3 (1:1000; Cell Signaling Technology, Danvers, Massachusetts, USA), and HRP-labeled goat anti-rabbit secondary antibody (1:5,000; Beyotime, Shanghai, China).

### 2.8. Stable Transfection

A complement sequence of mature miR-455-5p was synthesized and subcloned into the AgeI/EcoRI site of a GV280 vector (GeneChem, Shanghai, China) to generate a construct that overexpresses miR-455-5p. This construct was named LV-miR-455-5p. The empty vector (LV-miR-NC) was used as negative control. DU145 cells were transfected with LV-miR-455-5p or the control vector.

### 2.9. Tumor-Formation Assay

Animal experiments were undertaken in accordance with the National Institute of Health Guide for the Care and Use of Laboratory Animals and were approved by the ethics committee of Hospital Affiliated with Nantong University. Six 6-week-old immune-deficient BALB/C nu/nu male mice (Biomedicine-Tech, Nanjing, China) were each subcutaneously injected with DU145 cells (LV-miR-455-5p/LV-miR-NC, 10^6^ cells in 200 *μ*l medium) at two sides of dorsal scapular region.

### 2.10. Luciferase Assay

The 3′-UTR segment of CCR5 mRNA containing a miR-455-5p binding site was amplified by PCR using human DNA. The PCR products were cloned into the XhoI and NotI restriction sites downstream of the open reading frame of luciferase in the psiCHECK2 vector (Promega, Madison, Wisconsin, USA) to generate the CCR5 3′-UTR reporter. The binding site for miR-455-5p was deleted to generate the mutant reporter. For reporter assays, cells were transfected with psiCHECK2-CCR5-3′-UTR or mutation reporter plasmid and miR-455-5p mimics or NC. Luciferase activities were measured by dual-luciferase assays (Promega, Madison, Wisconsin, USA) 24 h after cotransfection.

### 2.11. Immunohistochemical Staining (IHC)

The paraffin-embedded xenograft tumors for IHC were processed in accordance with the manufacturer's instructions using a primary antibody: CCR5 mix (1:250; Protein Tech, Wuhan, China).

### 2.12. Statistical Analysis

All the above-mentioned experiments were repeated three times. The data in this study were presented as mean ± standard deviation. Statistical significance was calculated using Student's t-test or one-way ANOVA. P < 0.05 was considered statistically significant.

## 3. Results

### 3.1. The Diagnostic and Prognostic Value of miR-455-5p in Patients with PCa

**Figure 1:**
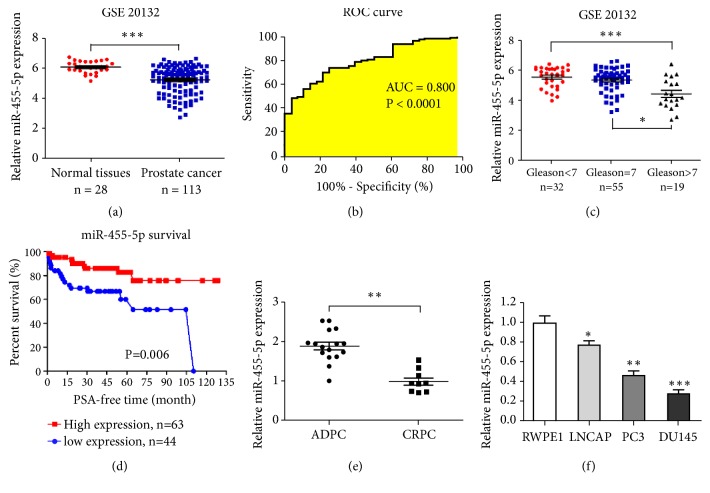
MiR-455-5p expression and effect on the diagnosis and prognosis of patients with PCa. (a) MiR-455-5p expression levels in patients of the GSE20132 database. (b) ROC curve analysis using expression levels of miR-455-5p in PCa and normal prostate tissues. (c) Reanalyzed data from GSE20132 showed that miR-455-5p expression level increased as the Gleason score ascended. (d) Kaplan–Meier curves of the RFS of patients with PCa stratified by the tissues of miR-455-5p levels. (e) Relative miR-455-5p expression in normal prostate cells RPWE1 and PCa cells LNCAP, PC3, and DU145. *∗*, P < 0.05; *∗∗*, P < 0.01; *∗∗∗*, P < 0.001.

We then reanalyzed the PCa database (GSE21032). Results showed that the miR-45-5p levels of the PCa tissues were significantly lower than those in the normal prostate tissues (6.06 ± 0.39 versus 5.52 ± 0.91, P < 0.0001; Figure 1(a)). ROC analysis demonstrated that miR-455-5p could discriminate between normal prostate tissues and PCa tissues (AUC = 0.800, P < 0.0001; Figure 1(b)). We then investigated the expression of miR-455-5p in PCa with different Gleason scores and discovered that the miR-455-5p expression level increased with increasing Gleason score (Gleason<7 versus Gleason=7 versus Gleason>7: 5.55 ± 0.68 versus 5.35 ± 0.79 versus 4.44 ± 1.02; Figure 1(c)). To further study the effect of mi-455-5p in PCa, we divided PCa samples into low- and high-expression groups in accordance with the relative miR-455-5p expression. The average number of 5.237 was used as cut-off for miR-455-5p expression. RFS analysis was performed in 107 PCa cases with follow-up data. Kaplan–Meier curves of RFS showed that patients with PCa and low miR-455-5p expression revealed shorter RFS times (P = 0.006; Figure 1(e)). Together, these results suggest that miR-455-5p may exert antioncogene functions in PCa progression.

### 3.2. MiR-455-5P Is Downregulated in PCa Cells

To study the role of miR-455-5p in PCa progression and determine the cell models for subsequent experiments, we detected the expression of miR-455-5p in the normal prostate cell line RPWE1 and the three PCa cell lines LNCAP, PC3, and DU145. Interestingly, compared with the normal prostate RPWE1 cells, three PCa cell lines with different malignancy degrees showed significantly low miR-455-5p levels. Among the three PCa cells, the androgen-receptor-positive cells of LNCAP which represent the low-malignancy cells showed the highest miR-455-5p level, whereas those of DU145, which represent high-malignancy cells showed the lowest miR-455-5p level. This result reinforced our prediction that miR-455-5p may act as an anti-oncomiRNA in PCa progression. To further study the effect of miR-455-5p on PCa progression, LNCAP and DU145 were chosen as cell models and subjected to cell functional assays subsequently.

### 3.3. MiR-455-5p Inhibits Cell Proliferation and Induces Apoptosis In Vitro

**Figure 2:**
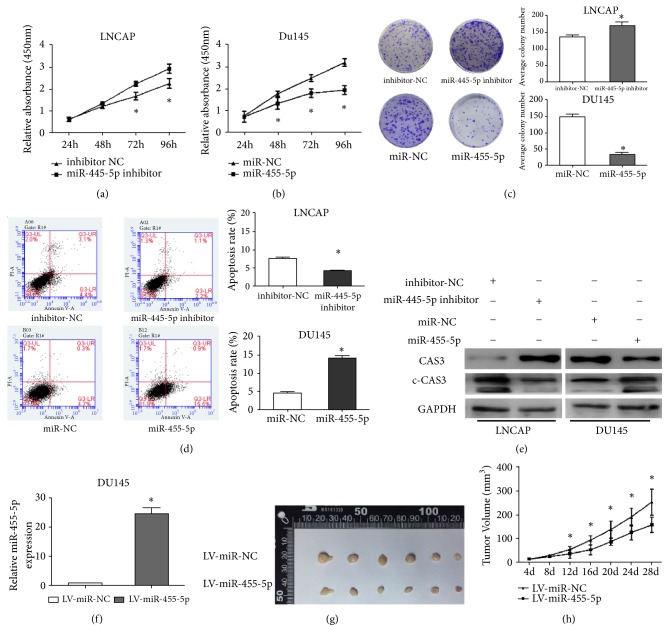
Biological functions of miR-455-5p on human PCa cell proliferation, cell apoptosis, and tumorigenicity. (a–c) Cell proliferation was assessed by CCK-8 and colony-formation assay in LNCAP and DU145 cells. (d) Cell apoptosis was analyzed by annexin V-FITC plus PI staining in both cells. (e) Western blot revealed the protein levels of caspase 3 and cleaved caspase 3 in LNCAP and DU145 cells 72 h after transfection. (f) After transfecting with LV-miR-455-5p virus, miR-455-5p expression was elevated compared with the negative-control group, as evaluated by qRT-PCR. (g) Image of tumor xenografts in nude mice injected subcutaneously with miR-455-5p-overexpressing DU145 cells. (h) Tumor volume was measured every 4 days. *∗*, P < 0.05.

Cell proliferation was assessed by CCK8 assays and colony-formation assay. MiR-455-5p inhibition increased the cell viability and colony numbers in LNCAP cells, whereas miR-455-5p overexpression decreased the cell viability and colony numbers in DU145 cells (Figures 2(a)–2(c)). Afterward, we examined cell apoptosis by the annexin V-FITC plus PI staining. As revealed by statistics, inhibiting miR-455-5p alleviates the cell apoptotic rate in LNCAP cells, whereas overexpressing miR-455-5p could induce the cell apoptotic rate significantly in DU145 cells (Figure 2(d)). To further investigate the mechanism of apoptosis induced by miR-455-5p, we applied Western blot to detect the expression level of caspase 3 and cleaved caspase 3 after treatment with miR-NC/miR-455-5p or inhibitor-NC/miR-455-5p inhibitor, respectively, in the two cell lines. We found that the miR-455-5p-directed regulation of cell apoptosis was accompanied by the cleavage and activation of caspase-3 (Figure 2(e)).

### 3.4. MiR-455-5p Inhibits PCa Cell Tumorigenicity In Vivo

For additional evidence on the tumor-suppressor role of miR-455-5p, we performed a xenograft assay after establishing stably miR-455-5p/miR-NC-overexpressing DU145 cells. The expression of miR-455-5p in LV-miR-455-5p/LV-miR-NC DU145 cells were verified (Figure 2(f)). The stably miR-455-5p-overexpressing cells significantly reduced xenograft growth and tumor volume relative to that in the negative-control group (Figures 2(g) and 2(h)). These data verify that miR-455-5p can inhibit tumor growth in vivo.

### 3.5. CCR5 Is a Direct Target of miR-455-5p

**Figure 3:**
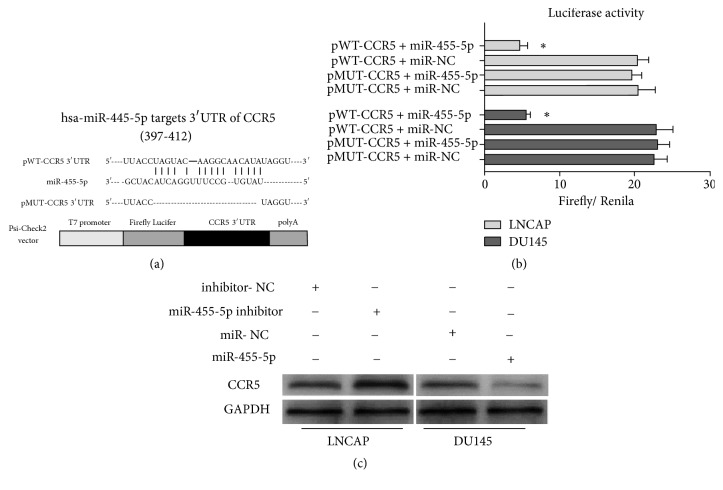
CCR5 as a direct target of miR-455-5p. (a) Diagram of putative miR-455 binding sites in the 3′-UTR (397–412) of CCR5 mRNA. (b) Relative activities of luciferase reporters containing CCR5 3′-UTR variants cotransfected with miR-455-5p or negative-control mimics in LNCAP and DU145 cells. (c) Protein levels of CCR5 72 h after miR-455-5p and inhibitor transfection in LNCAP and DU145 cells. *∗*, P < 0.05; *∗∗*, P < 0.01; *∗∗∗*, P < 0.001.

We performed bioinformatics analysis to explore the potential targets of miR-455-5p using the website microRNA.org (http://www.microrna.org/) and Target-Scan (http://www.targetscan.org). We found that miR-455-5p could bind to target sequences located in the 3′-UTR of CCR5 mRNA (Figure 3(a)). To check if a direct interaction was involved between miR-455-5p and CCR5, we performed luciferase reporter assays. After we cotransfected miR-455-5p with psi-CHECK2-WT-CCR5 or psi-CHECK-MUT-CCR5 in LNCAP and DU145 cells, we found that the cotransfection of miR-455-5p with wild-type reporters significantly decreased the luciferase activity (Figure 3(b)). Moreover, we performed Western blot analysis for CCR5 expression in the LNCAP and DU145 cells. The CCR5 expression level increased with miR-455-5p inhibition but decreased with miR-455-5p overexpression relative to those in the negative controls (Figure 3(c)). These results suggest that CCR5 is a direct target of miR-455-5p in PCa. However, additional cell functional assays with CCR5 reinhibition or restoration are necessary to confirm whether miR-455-5p suppresses PCa progression through CCR5 mediation.

### 3.6. MiR-455-5p Regulated Cell Proliferation and Induced Apoptosis by Targeting CCR5

**Figure 4:**
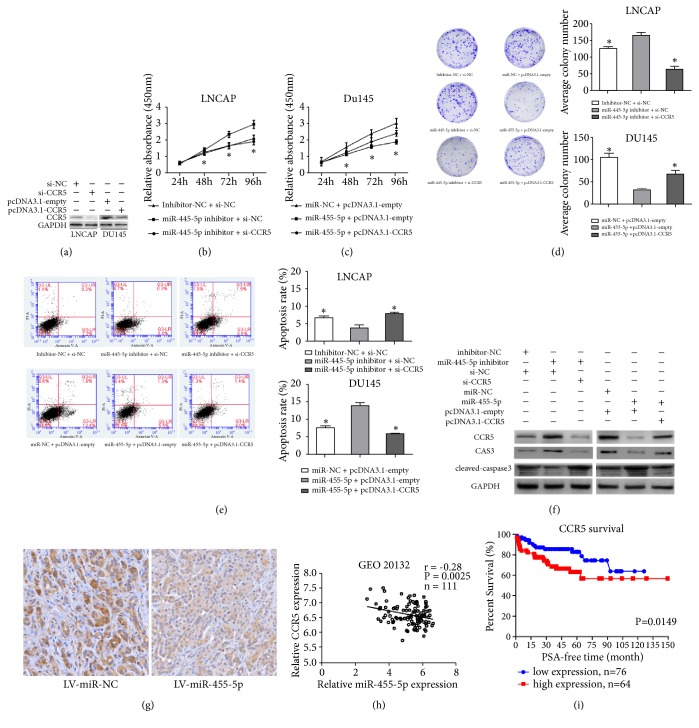
Regulation of cell proliferation and apoptosis by miR-455-5p through directly targeting CCR5. (a) Protein levels of CCR5 in LNCAP and DU145 cells 72 h after transfection. (b–d) Cell proliferation was assessed by CCK-8 and colony-formation assays in LNCAP and DU145 cells after cotransfection. (e) Cell apoptosis was analyzed by annexin V-FITC plus PI staining in LNCAP and DU145 cells. (f) Western blot revealed the protein levels of caspase 3 and cleaved caspase 3 and CCR5 in LNCAP and DU145 cells 72 h after cotransfection. (g) Representative images of IHC for CCR5 on xenografts. Brown signals on the cell membrane represented CCR5 expression level (Magnification, ×200 bar). (h) Inverse correlation of CCR5 and miR-455-5p in the GSE20132 database (r = −0.28, P = 0.0025). (i) Kaplan–Meier curves for the RFS of patients with PCa stratified by tissue CCR5 levels. *∗*, P < 0.05.

Primarily, we examined the CCR5 expression level by transfection with si-NC/siCCR5 in LNCAP and pcDNA3.1-empty/pcDNA3.1-CCR5 in DU145 cells (Figure 4(a)). Cell proliferation was assessed by CCK8 assays and colony-formation assays on the basis of miR-455-5p inhibition; CCR5 knockdown with siRNA decreased the cell viability and colony number in LNCAP cells. By contrast, on the basis of miR-455-5p overexpression, CCR5 restoration by plasmid vector rescued the cell viability and increased the colony number in DU145 cells (Figures 4(b)–4(d)). These results suggest that miR-455-5p regulated cell proliferation at least in a CCR5-mediated manner. In the following apoptosis assays, we found by inhibiting miR-455-5p that CCR5 knockdown with siRNA could promote the cell apoptotic rate in LNCAP cells. By contrast, CCR5 supplementation followed by miR-455-5p overexpression alleviated cell apoptosis in the DU145 cells (Figure 4(e)). Previously published articles reported that CCR5 blockage induces cell apoptosis by activating and cleaving caspase 3 [16]. Accordingly, we examined the protein level of CCR5 and subsequently caspase 3 and cleaved caspase 3 in different transfection modes. We discovered that CCR5 could attenuate the cleavage and activation of caspase 3 induced by miR-455-5p overexpression, which means that miR-455-5p could promote cell apoptosis at least in a CCR5-mediated manner (Figure 4(f)). To further confirm the direct target CCR5, we performed IHC staining for CCR5 in the xenografts harvested from tumor-formation assay. MiR-455-5p overexpression reduced the CCR5 level as expected (Figure 4(g)). Overall, we verified that miR-455-5p regulated cell proliferation and colony formation, as well as induced apoptosis, in PCa by directly targeting CCR5.

### 3.7. Correlation between CCR5 and miR-455-5p in the Database and the Prognostic Value of CCR5

To consolidate the regulatory relationship between miR-455-5p and CCR5, we reanalyzed the expression of miR-455-5p and CCR5 in the GSE20132 database. As predicted, an inverse correlation was found between the expression levels of miR-455-5p and CCR5 (Figure 4(h)). Although studies have revealed the vital role of CCR5 in PCa, we divided PCa samples into low- and high-expressing groups using the average expression number 6.570. RFS analysis was performed in 140 PCa cases with follow-up data. Kaplan–Meier curves of RFS showed that patients with PCa and high CCR5 expression attained shorter RFS times (P = 0.0149; Figure 4(i)). This result was in contrast with the miR-455-5p in PCa (Figure 1(e)). These two results confirmed that miR-455-5p suppresses the PCa progression at least by directly targeting CCR5.

## 4. Discussion

Exploring the underlying mechanism of prostate carcinogenesis remains critical for early diagnosis, predicting prognosis, and effective therapeutic strategy. To date, numerous miRNAs have been discovered and verified to act as tumor suppressor or oncogene in PCa progression.

MicroRNA-455-5p is located at chromosome 9:114, 209, 434–114, 209, 529 and has been validated to play an essential role in various cancers. Moreover, studies have shown that miR-455-5p is involved in multiple biological and pathological processes, such as cell proliferation, apoptosis, migration, and invasion. For instance, miR-455-5p has been verified to successfully suppress cell viability and induce cell apoptosis by targeting the RAF proto-oncogene serine/threonine protein kinase (RAF1) in colorectal cancer [8]. Moreover, miR-455-5p was reduced significantly in gastric cancer cells and could inhibit human gastric cancer-cell proliferation and invasion, as well as promote cell apoptosis, by targeting a member of the RAS oncogene family (RAB18) [11]. Moreover, low miR-455 expression was associated with poor prognostic features (multiple tumor nodes, high Edmondson–Steiner grading, advanced tumor-node-metastasis (TNM) stage, and venous infiltration) and could significantly suppress the migration and invasion of hepatocellular cancer [10]. In short, miR-455-5p serves as an anti-oncomiRNA in the above-mentioned cancers. However, the expression and mechanisms of miR-455-5p in regulating human PCa progression remain unclear.

In this study, reanalysis of the data obtained from MSKCC showed that miR-455-5p was poorly expressed in PCa. We demonstrated that miR-455-5p can distinguish PCa from normal prostate tissues. In particular, the specimens with high Gleason scores exhibited low miR-455-5p expression, whereas those with low miR-455-5p expression exhibited shorter RFS times than those with high-expression levels. We observed that miR-455-5p expression was higher in the low-malignancy cell line (i.e., LNCAP) than in the high-malignancy cell lines (i.e., PC3 and DU145). These results suggest that miR-455-5p may participate in PCa progression, and miR-455-5p can be used as a biomarker for diagnosis and prognosis analysis.

Subsequent functional assays showed that the knockdown of miR-455-5p with an inhibitor promoted the cell proliferation and avoided apoptosis in LNCAP cells, whereas overexpression of miR-455-5p suppressed cell proliferation and triggered apoptosis in DU145 cells. These results revealed miR-455-5p acts as an anti-oncomiRNA in PCa. Through reanalysis of the GEO20132 database, we detected that CCR5 attained a higher level of expression in the PCa tissues than in the normal prostate tissues. Patients with higher mCCR5 expression exhibited shorter RFS times, a result which differs from that with miR-455-5p. Through correlational analysis, we found an inverse trend between miR-455-5p and CCR5 levels.

CCR5, also known as CD195, is a family of structurally related proteins initially recognized as mediators of chemotaxis and cellular homing [17]. To date, CCR5 has been demonstrated as involved in various biological processes, including PCa progression. The C–C motif chemokine ligand 5 (CCL5) mediated its biological activities by activating CCR5. The CCL5/CCR5 axis activates protein kinase Cdelta (PKCdelta), c-Src, and hypoxia-inducible factor-1 alpha (HIF-1alpha) signaling cascades to induce vascular-endothelial-growth-factor-dependent angiogenesis [18]. According to previously published articles, CCR5 knockdown through siRNA action or antagonist blockage could suppress metastasis and inhibit cellular proliferation in PCa cells [19, 20], especially inducing cell apoptosis by activating and cleaving caspase 3 [16]. Interestingly, this notion conforms to our finding that miR-455-5p-induced PCa cell apoptosis is accompanied by the downregulation of caspase 3, along with the upregulation of cleaved caspase 3.

To define the regulatory mechanism within miR-455-5p and CCR5, we consulted two online databases. Through bioinformatics analysis, we predicted the oncogene CCR5 as a potential target for miR-455-5p. We then validated the regulation of miR-455-5p on CCR5 by luciferase reporter assay and Western blot analysis and found that miR-455-5p was a negative regulator of CCR5. Moreover, we experimentally verified that CCR5 restoration notably mitigated the suppressive effect of miR-455-5p on PCa cell proliferation and apoptosis. IHC performed on xenografts confirmed that CCR5 was inhibited by miR-455-5p in vivo.

Given our discoveries, we speculate that miR-455-5p may be a potential therapeutic molecule for PCa treatment and acts by regulating CCR5. However, the exact mechanism by which miR-455-5p affects PCa cell phenotype requires further study.

In summary, this study revealed that miR-455-5p expression is frequently decreased in PCa cells and tissues. Our work also demonstrated the tumor-suppressive function of miR-455-5p in significantly suppressing proliferation and triggering the apoptosis of PCa cells. In addition, we experimentally identified CCR5 as a direct and functional target of miR-455-5p. Our data highlighted that miR-455-5p is a potential tumor suppressor and may be a novel therapeutic target for PCa treatment.

## Data Availability

The data used to support the findings of this study are included within the article.
